# Role of Stress in the Origin of Life

**DOI:** 10.3390/life12111930

**Published:** 2022-11-18

**Authors:** Vladimir Kompanichenko, Oleg Kotsyurbenko

**Affiliations:** 1 Institute for Complex Analysis of Regional Problems RAS, 679016 Birobidzhan, Russia; 2 Institute of Oil and Gas, School of Ecology, Yugra State University, 628012 Khanty-Mansiysk, Russia; 3 Network of Researchers on the Chemical Evolution of Life, Leeds LS7 3RB, UK

**Keywords:** origin of life, population of microorganisms, hydrothermal system, oscillations in parameters, prebiotic microsystem, stress

## Abstract

The article shows the compatibility of the concept of thermodynamic inversion (TI) of the origin of life with the theory of stress in (micro)biology. According to the proposed TI concept, the first microorganisms on Earth were formed through an effective (intensified and purposeful) response of organic microsystems to incessant oscillations of physicochemical parameters (i.e., to periodic stress) in a hydrothermal environment. This approach allows us to explain the ability of contemporary microorganisms to respond to stress at the individual and population levels. The ability of microorganisms to effectively react to environmental stress factors is corroborated by a number of molecular and other mechanisms that are described in the article.

## 1. Introduction

Over the past few decades, prebiotic chemistry has achieved significant success. The initial models, such as coacervates [1] or proteinoid microspheres [2], were quite simple. Now, many of the modern models created and studied within the framework of contemporary prebiotic chemistry are much more complicated. In particular, there are some prebiotic models based on a combination of RNA and lipid vesicles, in which the latter mimic the cellular cytoplasmic membrane [3,4,5]. Another version of such models is based on the introduction of pre-DNA and pre-protein into lipid vesicles with their subsequent self-replication [6,7].

The culmination of laboratory experiments in the field of prebiotic chemistry is the production of “protocells” and the more complex “artificial cells”. Many of them bear some resemblance to living microorganisms. Such models can exhibit catalytic activity and the ability to divide, such that the chains of their nucleic acids can reproduce themselves. However, it should be borne in mind that the DNA chains that are spontaneously synthesized in the models are abiotic and do not contain biological information for billions of years accumulated in the genes of living microorganisms. Sometimes, experimenters designate the processes in such laboratory models as “almost life”, although the above-mentioned initial signs of a living state are not self-sustaining, and we therefore cannot consider them biological.

So far, no laboratory experimenters have obtained a population of actually living subcells from prebiotic microsystems, which are characterized by the following key biological features: (1) an extremely high rate of biochemical reactions; (2) behavior adequate to certain environmental conditions or changes, that is, purposeful behavior; (3) closes interaction between cells, both within cell populations and between cell populations in the community. Even advanced scenarios of prebiotic evolution do not explain how populations of microbial cells became active with respect to the environment and how they acquired a tendency towards expansion. This apparent gap between prebiotic models and living cells is a “mysterious leap” in evolution or a missing link [7]. The three modern approaches to the origin of life—complex (or primary metabolism), the RNA world (or primary gene), and the cellular (universal common ancestor)—do not explain the nature of this missing link.

An attempt to consider the nature of the gap between nonliving prebiotic microsystems (or protocells) and living cells on the basis of a different approach was undertaken within the framework of the concept of thermodynamic inversion (TI), or simply the inversion concept of the origin of life [8,9,10,11,12,13]. It differs from other concepts in that it postulates the need for an effective response of a prebiotic system (a cluster of organic microsystems) to oscillations of physicochemical parameters in the hydrothermal environment. Oscillations should occur with pauses in the high-frequency range, reaching their extreme values from time to time. Such periodic changes are stress factors for prebiotic systems. If the reaction of clusters of organic microsystems to such external influences as periodic stress becomes intensified and purposeful (which is possible under the conditions described below), they transform into the simplest living systems—primary populations of subcells.

The purpose of this article is to correlate the theoretical concepts of TI with respect to the role of periodic stress in the emergence of initial sub-microorganisms from organic microsystems, on the one hand, and modern factual data on the role of stress for the existence and development of a population of microorganisms, on the other.

## 2. Main Provisions of the Inversion Concept of the Origin of Life

The proposed concept of thermodynamic inversion (TI) focuses on the key moment of nonliving prebiotic microsystems’ transition to the first life forms (subcells). Its main theses are summarized in this section. A detailed presentation of the latest version of the inversion concept is given in other works [11,12].

The meaning of thermodynamic inversion as a key thermodynamic transformation during the transition of a prebiotic system to a living state is as follows. It is well known that inanimate natural systems eventually evolve in the direction of increasing entropy, which follows from the second law. It is also known that biological evolution paradoxically proceeds in a thermodynamically opposite (negentropic) direction—with an increase in free energy and information in the system due to a relative decrease in entropy. Within the framework of the concept of thermodynamic inversion, attention has been drawn to the fact that the presence of these contradictory trends means the transition of a non-living chemical system from existence under conditions of the prevailing contribution of entropy to existence under conditions of the prevalence of the contribution of free energy and information at the moment (or a short period) of the appearance of living state in it, regardless of the constituent molecules. This transition is called thermodynamic inversion, or thermodynamic reversal. It is as if we observe a car moving first in one direction and then in the opposite direction; in that case, we understand that it has turned around somewhere (even if we did not see it). After thermodynamic inversion, the transformed system can no longer exist as an ordinary chemical system. It must build its internal chemical and information processes, as well as interaction with the environment, in such a way as to generate more free energy than can be neutralized by entropy. This way of organizing the system can be designated as “negentropic”, and it occurs at the moment of the origin of life. From the point of view of the TI concept, chemical evolution in itself, as a process of the complication of organic matter, is insufficient for the origin of life, since it does not include the transition of the system to the qualitatively different (negentropic) thermodynamic state. This requires a prebiotic chemical revolution—a combination of the presence of evolving organic systems of optimal composition (which has yet to be clarified) and thermodynamic inversion. During the latter, the system is periodically exposed to stress factors (changes in the environment), and through an effective stress response, it builds a negentropic network of (bio)chemical reactions and information processes aimed at maintaining and developing such a state.

**Figure 1:**
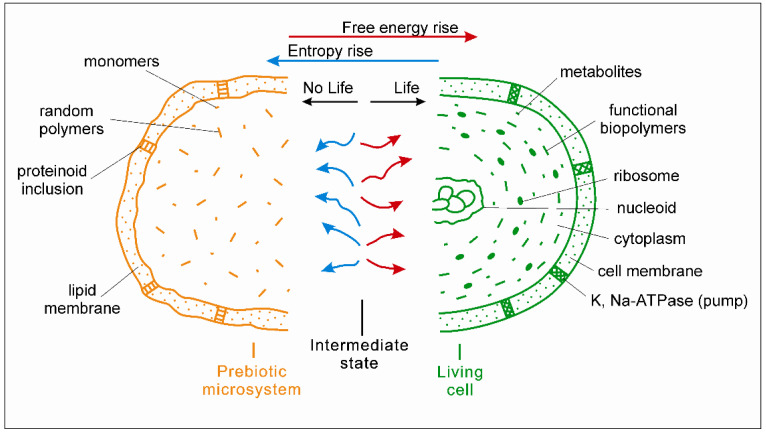
Scheme of change in the thermodynamic state of an inanimate prebiotic microsystem during its transition to the primary living state through an intermediate state.

The transformation of a cluster of nonliving prebiotic microsystems into a primary population of living subcells takes place at the moment of thermodynamic inversion, when the system obtains the total contribution of free energy prevailing over the contribution of entropy (in the same way as the contribution of information becomes prevailing over the contribution of information entropy). An inanimate prebiotic system passes into the primary form of life (a living subcell) through a thermodynamically intermediate state, which is schematically shown in Figure 1. The optimal composition of prebiotic microsystems most suitable for TI should be clarified by the joint efforts of experimenters and theorists involved in the study of the origin of life. As an option, a model of a three-dimensional organic microsystem was previously proposed, consisting of the main biologically important molecules—lipids, polyamino acids (proteins), and nucleotides [11]. However, consideration of this model is not the purpose of this article.

According to the TI concept, during the transition to life, prebiotic systems are in conditions far from equilibrium, which are initiated by incessant oscillations in physicochemical parameters in the environment, such as pressure, temperature, concentrations of components, and electric potential. An important factor is the periodic change in the scale of oscillations, which leads to a periodic replacement of extreme conditions by pauses. Such regimes are typical of hydrothermal systems, but not typical of the ocean or ice layers. Therefore, it is the hydrothermal fluid that migrates in the Earth’s crust up to the surface or ocean floor, which is considered the most suitable area for the emergence of life.

On the left in the Figure 1, in yellow, is a non-living prebiotic microsystem (its half) composed of random polymers, monomers, and simple molecules. In the center is a thermodynamically intermediate state of the microsystem between non-life and life, with relative equality of contributions from chemical reactions producing free energy (red) and entropy (blue). On the right, in green, is a primary living microorganism (its half). The picture shows its evolutionarily advanced form: a modern prokaryotic cell with basic cellular structures, including a nucleoid (circular DNA), ribosomes, and a cell membrane.

**Figure 2:**
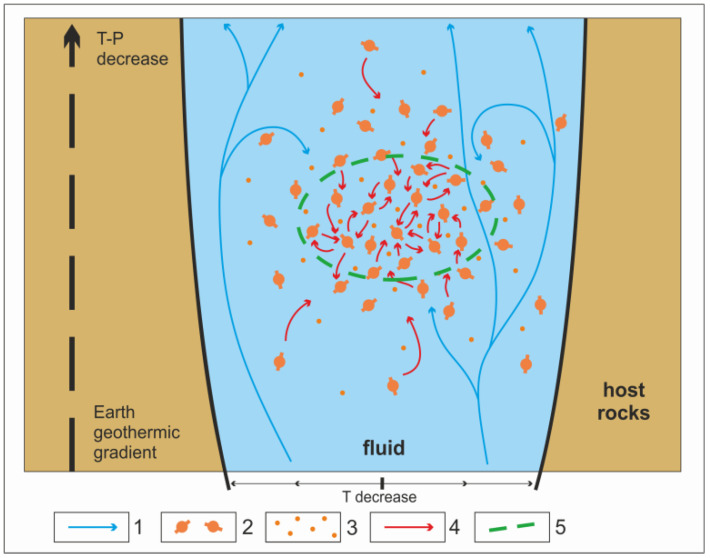
Scheme of the conversion of a cluster of prebiotic microsystems into initial population of living subcells in the upper zone of hydrothermal channel: 1—laminar and convective fluid currents to the surface; 2—interacting organic assemblies (microsystems), composed mainly of lipids, proteins, and nucleotides; 3—disseminated organic molecules; 4—directions of free energy transfer; 5—primary population of living subcells.

Under the described nonequilibrium conditions, thermodynamic inversion occurs due to the enhanced and targeted response of microsystems to high-frequency oscillations (“pumping”) of the physicochemical parameters of the environment. Clusters of organic microsystems consisting of lipids, polyamino acids, and other components are formed in the ascending hydrothermal flow due to self-assembly. Continuous interaction between microsystems is supported by different-mode oscillations in the environment (Figure 2). It is well known that abrupt changes occurring in chemical systems under highly nonequilibrium conditions induce extremely fast chemical reactions [14]. Their role in responding to external influences in connection with the process of the beginning of life remains to be studied. The thermodynamic inversion of a cluster of organic microsystems existing in an oscillatory mode occurs in the upper part of hydrothermal channels. Here, the environmental conditions become less extreme (the absolute values of pressure and temperature decrease, as well as the scale of physicochemical fluctuations). Subsequently, the primary living systems that are formed—i.e., populations of the simplest subcells—move out into the ocean, where their biological evolution continues. The proposed mechanism for the emergence of life through thermodynamic inversion with certain variations can be common for different inhabited planets, since the basic terms used—entropy, free energy, and information—are the most fundamental attributes of the Universe.

A laboratory experiment scheme is proposed to test this approach. It consists of studying the transformations that take place in prebiotic microsystems of different types when they are “pumped” by oscillations of physicochemical parameters of different ranks, including pressure, temperature, pH, Eh, electric potential, etc. [12]. In the course of such experiments, it is necessary to reveal the presence of such a reaction of organic microsystems to physicochemical “pumping”, which brings them closer to a living state (strengthening the role of active transport, increasing the degree of homochirality, etc.).

Let us emphasize the main difference between the proposed TI concept and existing alternative concepts. In the latter concepts, it is assumed that living systems arose at some stage of chemical (prebiotic) evolution directed at the consistent chemical complication of organic microsystems (microstructures), with different variations. The need for TI is not provided for in them. According to the approach outlined in the article, it is at the moment of thermodynamic inversion that active metabolic processes are launched in prebiotic microsystems that were inactive earlier (with respect to the environment). According to this understanding, biochemical reactions do not arise by themselves in the course of chemical prebiotic evolution. They are instead launched and integrated through the effective response of a cluster of organic microsystems (or a separate microsystem) to continuous oscillations of physicochemical parameters in the environment, occurring in an optimal mode for the emergence of life.

This difference can be illustrated by the following example. There is a large group of microorganisms belonging to the domains of Archaea and Bacteria living in harsh environments such as extreme temperature and/or pH or high concentrations of metals and/or salt. Such microorganisms are also capable of responding to sudden changes in environmental conditions. A group of Italian scientists has studied and generalized the molecular mechanisms responsible for the survival and adaptation of this type of microorganism, which is related to thermophilic species. The main focus was on their adaptation to toxic metals, with particular emphasis on As (V), As (III), and Cd (II) [15]. The four main mechanisms of heavy metal resistance were found in these microorganisms: (1) an extracellular barrier, forming a selectively permeable system; (2) efflux of metal ions; and (3) enzymatic reduction of metal ions; and (4) intracellular sequestration by small molecule complexing agents or metal-chelating proteins. In more complex mesophilic microorganisms, some of the mechanisms described are already absent. The thermophilic Archaea and Bacteria are known to be very primitive and are at the base of the phylogenetic tree of life on Earth [16,17]. Within the framework of the inversion concept, these facts can naturally be interpreted as follows. At the earliest stages of biological evolution, microorganisms developed their defense mechanisms by effectively resisting extreme environmental conditions. In mesophilic microorganisms, the need for some of the previously formed defense mechanisms had already disappeared, since they existed and exist in less extreme conditions. If we consider these facts in the framework of the concepts of the emergence of life as a result of only chemical evolution without the manifestation of thermodynamic inversion, it becomes unclear at the expense of what and how these protective mechanisms developed in thermophilic microorganisms. At least in scenarios of the origin of life through sequential chemical evolution, such facts are not taken into account, and, accordingly, they do not explain them.

## 3. Correlation between the Inversion Concept and the Stress Theory in Biology

The TI concept correlates with the fundamental theses of biology in the field of the stress theory, the founder of which is Hans Selye [18]. The key points of the stress theory can be summarized as follows. External influences on a living organism (positive or negative effect of environmental stress factors) cause tension in it (stress itself), followed by a response to stress. Moderate stress factors are necessary to maintain the vitality of the organism, as they allow it to develop an effective response to stress (“stress response”) that overcomes environmental pressure. The action of very strong stress factors (distress) leads to the degradation of the organism. Lack of exposure to stress factors (stress = 0) also initiates the degradation of the organism in the long run.

The inversion concept explains these regularities as follows. The fluctuations of the physicochemical parameters of the environment discussed above affect a prebiotic microsystem in the same way as the stress factors of the environment on a living microorganism. When exposed to moderate (optimal) force, the prebiotic microsystem has the ability to develop an enhanced and targeted counteraction (effective stress response), which can transform it into a primary form of life. In the case of a very strong external influence, the prebiotic microsystem is under the overwhelming pressure of entropy (weakened stress response) and cannot be transformed into the primary form of life. In the absence of an external influence (absence of environmental stress factors), a stress response does not develop in the microsystem at all. It also does not have the ability to transition to life.

As a result of fluctuations in physicochemical parameters in the medium, the degree of external pressure (thermodynamically related to the flow of entropy) on the primary form of life will vary all the time. If the environmental pressure begins to exceed the ability of the primary (sub)microorganism to actively exist, then it is forced to return to its original prebiotic state. However, such a reverse transition should initiate the counteraction of an already incipient living microsystem that possesses the sparks of a purposeful, survival-oriented response. It can be assumed that in such conditions of arising “distress”, the primary form of life will purposefully reorganize its internal structure and functions in such a way as to survive an unfavorable period and preserve the possibilities for transition to active existence when external pressure subsides. Such “passive” resistance to external pressure can be compared with the state of suspended animation, which is studied by modern microbiology [19,20,21,22,23]. Consequently, it follows from the considered approach to the origin of life on Earth that the microorganism could have initially existed in two states: a) passive, if the “pressure” from the environment (the action of stress factors) exceeded its ability to effectively counteract; b) active, if its response to environmental stressors was enhanced and targeted.

Below are three main provisions arising from the inversion concept, which correlate with the fundamental knowledge of modern microbiology.

1. A microorganism is able to pass from a passive state (suspended animation) to an active state (free living form) and vice versa. The current state depends on the conditions in the environment (its “pressure”, which within the framework of thermodynamics can be expressed through flow of entropy) and the internal resources of the microorganism.
2. When unfavorable conditions in the environment are approaching, a free-living microorganism is able to purposefully rebuild its structure and functions, consistently preparing for the transition to a passive state (suspended animation) to maintain potential viability.
3. To get out of suspended animation, the (resting) microorganism must receive an impulse (exposure to a stress factor) from the environment, indicating a decrease in the external pressure. In response to this impulse, conserved vital functions are activated, and many reverse sequential transformations are triggered, ensuring its transition to an active state.

## 4. Microbial Stress Responses

The evolutionary history of the Earth, especially its early period, was associated with various cataclysms, accompanied by a continuous change in geological, water, and atmospheric conditions. During these global transformations, life originated on the planet. In the process of evolution, ancient microorganisms developed mechanisms to resist the adverse environmental factors inherited by subsequent generations of life forms.

Sudden unfavorable changes in conditions either lead to cell death or activate powerful adaptation mechanisms, including damage repair mechanisms, in particular repair of DNA damage, and global reorganization of metabolism. All these transformations are associated with large time and energy resources. This indicates that cells exposed to adverse factors are under stress [24,25].

Moreover, in reality, bacteria, irrespective of natural habitat, are exposed to constant fluctuations in their growth conditions which are usually not optimal. Variations in any environmental parameters can affect the maximum growth rate and, thus, can represent an environmental stress for the microbe. As a result, most bacteria live in a constant state of stress. Furthermore, some microorganisms (extremophiles) view stresses such as extremes in pH or temperature as a lifestyle choice. The ability of microbes to sense and respond (correctly) to such alterations in the environment is crucial to their survival.

The global activation and rearrangement of cellular metabolism in response to stress factors are controlled by the corresponding genes and are accompanied by a global change in the type of gene expression involving cellular hormones called alarmones, which trigger a cascade of sequential reactions using signal transduction systems initiating stress-responsive pathways, some of them being very conserved.

The response to the imposed stress is accomplished by changes in the patterns of gene expression for those genes whose products are required to combat the deleterious nature of the stress. The up-regulation of the transcription of stress responsive genes is achieved by the activation of transcription factors that interact with RNA polymerase to co-ordinate gene expression. One family of transcription factors that play a role in stress resistance is a subunit of RNA polymerase, the sigma factor, which is essential for initiation transcription, playing a key role in promoter recognition. The environmental stress response is controlled by a supramolecular complex known as the stressosome.

The cell is forced to synthesize those enzymes that are necessary under stressful conditions, and hence, genes encoding such enzymes must be activated. Mobile genetic elements (MGE, in prokaryotes these are conjugative plasmids, transposons, and integrons) are involved in the activation of the genetic regulatory system of the organism [26]. MGE is a molecular tool by which the genome can be considered as a special cellular organ designed to detect deviations in the cell from its normal functioning. Moreover, as a result of such detections, the genome can reorganize itself, depending on the needs of the cell under specific conditions. In general, the genome can respond to stress by inducing interspecific transfer of MGE. In this case, the organism experiences the so-called “genomic shock” and the subsequent restructuring of the genome. Plasmid DNA is capable of independent reproduction in the cell and has a special machinery for its transfer to neighboring cells (lateral or horizontal gene transfer), which may cause the formation of new biological species. At the same time, under normal environmental conditions and cellular homeostasis, organisms developed a mechanism for controlling the transfer of MGEs.

Thus, bacteria have developed stress responses, which aim to temporarily increase tolerance limits. These stress responses are often very specific, each specialized for a particular kind of stress. Depending on the nature of the stressor and the type of the damage caused, the cell response may be different. Some stress responses facilitate bacterial transition from a free-living organism to a host-invading pathogen. Bacterial adaptive responses include the development of spores and competence, the activation of motility to more favorable locations, the synthesis of antibiotics and proteases, and changes in energy production systems. The fine-tuning of respiratory electron transfer routes and energy coupling mechanisms play important roles in the ability of bacteria to cope with variations in oxygen and nutrient supply.

The main goal of adaptation mechanisms is to maintain homeostasis in a cell compensating a negative influence of stress factors. For these purposes, the cell synthesizes heat and cold shock proteins and performs a temperature-dependent change in the degree of lipid unsaturation (temperature stress). It uses osmoprotectants and mechanisms of osmoregulations such as K^+^ ion influx/glutamate-biosynthesis-coupled systems (osmotic stress) and the modulation of the primary proton pumps as well as the K^+^/H^+^ and Na^+^/H^+^ antiporters (acid tolerance), and activates mechanisms of prevention of transporting harmful compounds such as xeno- or antibiotics in the cell. In an oxidative stress response, (2Fe-2S) enzyme centers are usually involved as sensors changing their oxidation state when challenged with oxidative stress. Fe and sulfur compounds are considered to be important components in the metabolic system of the last universal common ancestor (LUCA). This illustrates an ancient nature of some of stress response systems in microorganisms. Another indication of the evolutionary relation of general stress responses is the stress-dependent sigma factor regulating transcription and probably operated in the original ancestor.

In facultative aerobic microorganisms, the transition between aerobic and anaerobic metabolism is accompanied by alterations in the rate, route, and efficiency of pathways of electron flow. The catabolism of fuel molecules is associated with the reduction of NAD^+^ to NADH. During the transition to oxygen limited growth and an increased level of NADH built up, as it is less efficiently reoxidized to NAD^+^ as a result of reduced aerobic respiration. These roles of NADH and NAD^+^ provide a link between energy homeostasis and gene regulation.

Furthermore, microorganisms can control the production of the various respiratory pathway enzymes in response to the availability of alternate electron acceptors. When several e-acceptors are present simultaneously, the more energetically favored acceptor will be used first. Such diversity of metabolic pathways is also an adaptation of a cell to unfavorable conditions. Thus, stress activates the defense mechanisms of the cell, which in turn lead to a rearrangement of the cell metabolism and, often, to a decrease in its level, which contributes to the survival of the cell in conditions that do not allow balanced metabolic activity.

One of the mechanisms of adaptation to stress is the dissociation of bacteria—the splitting of a homogeneous population into variants that differ in morphological, physiological, biochemical and biological properties. The properties of different species and even strains may be different. This creates a phenotypic variety of forms on a single genetic basis. Species that are most adapted to specific environmental conditions survive and develop. The described dissociation is especially typical for pathogenic bacteria. They rearrange their genetic apparatus using various sensory and regulatory mechanisms as a response to their transition to the external environment from a human or animal organism, or after a sharp change in the environmental conditions. This allows them to maintain their viability and to change their virulence and antigenic properties.

Thus, biosystems of various levels of organization can be subject to stress. Accordingly, the methodological approaches to studying the effect of stress and the response of biosystems to it are also different [27]. At the level of the microbial community, the methodology includes incubation experiments with varying physicochemical parameters, the use of various inhibitors and selective substrates to identify the potential of a particular microbial group, and the use of radioactive substrates to study the pathways of decomposition of organic matter [28]. At the microorganism level, experiments include cultivation, the study of enzymatic activity, and genomic and transcriptomic studies and regulatory mechanisms of gene expression under various conditions, followed by bioinformatic processing of the obtained data array and interpretation of the results [29,30]. Shifts in community composition occur due to different biogeochemical capabilities of organisms to resist stress. Thus, the influences of all types of stress operate at both physiological and community composition levels with a linkage between environmental conditions and biogeochemical processes. While the physiological effects likely regulate short-term responses of soil communities and processes, shifts in community composition are likely to regulate them over longer periods [31].

Microbial community is a complex biological system consisting of different microbial groups trophically connected to each other [27]. To cope with stressors in the environment, the microbial community should maintain its basic functional structure according to Le Chatelier’s law [27]. It rearranges the trophic interactions and the key microbial groups in order to compensate negative impacts of the environmental changes. It in turn results in redirection of organic matter flows in the community. For example, an anaerobic microbial community producing methane can change its main pathways of organic matter degradation at lower temperature and pH. As a result of such changes, either hydrogen-dependent and acetoclastic methanogenic archaea or homoacetogenic bacteria become the key terminal microbial group, resulting in different product composition.

The diversity of metabolic pathways and taxonomic groups in the community is an important mechanism for withstanding stressors and maintaining their function. The more extreme the external conditions, the less microbial diversity and the more difficult it is for the microbial community to maintain its functionality when the external conditions change, and, hence, the lower is its adaptive potential. Nevertheless, under quite stable conditions, a microbial system consisting of extremophilic microorganisms is formed. Its sustainability is primarily determined by specific conditions of the ecological niche, in which unique non-competitive adaptive survival mechanisms of extremophiles and their proper functionalities are in demand. Thus, microbial community has basic mechanisms of coping with environmental stress factors aiming at keeping its functionality in the environment.

An additional mechanism of resistance to stress can operate in associative mutually beneficial relations between bacteria and plants, for example, in the rhizosphere. In relation to xenobiotics, this mechanism results not only in increasing tolerance to the harmful compound, but also in its joint active removal from the environment [32].

Thus, microorganisms with their huge number in populations, have the mechanisms of physiological variation and transfer of genetic determinants developed by evolution and are in a state of constant adaptive progress in accordance with changing environmental conditions. Activation of the resistance mechanism system in microorganisms, some elements of which are related to very ancient mechanisms of stress response, occurs by signal transduction systems with the participation of transcription factors. These factors bind to specific sites in the DNA molecule and induce the expression of the genes responsible for the cell defense system. The result of this response is a global restructuring, both at the level of an individual cell and at the level of the community, and the activation of speciation processes as a mechanism for increasing the number of more adapted organisms to changing environmental conditions.

## 5. Conclusions

In a number of publications devoted to the elaboration of the TI concept [9,10,11,12,13], it was substantiated that chemical evolution in itself is insufficient for the emergence of primary life forms. This also requires short-term oscillations in physicochemical parameters in the environment, which exert periodic stress on prebiotic systems. When the stress response of these systems is effective (which is possible under certain conditions), they evolve towards life. From such an understanding of the process of the emergence of life, the conclusion follows that all subsequent (more complex) microorganisms must necessarily respond to stress, and in the event of an effective response, they achieve the opportunity for further development. Section 3 and Section 4 of this article show that the ability of microorganisms to respond to stress at the individual and population levels is indeed universal. This confirms the objectivity of the TI concept and its potential to explain the prehistory of the stress phenomenon observed in the modern world of microorganisms. Additionally, within the framework of this approach, the conclusion was substantiated that the main stages of the origin of primary microorganisms are fixed in the anabiotic cycle of modern bacteria and each time they are repeated during the exit of the bacterial population from the anabiotic state [13].

From this approach, it follows that there is a need to move to the next stage of laboratory research on the problem of the origin of life. So far, numerous experiments in prebiotic chemistry have been carried out mainly under stable conditions. Some experiments, which are summarized in [12], were carried out under conditions of reversible oscillations of physicochemical parameters (temperature, humidity) in the medium. These works have demonstrated the progressive complication of organic macromolecules under oscillatory conditions, in comparison with stable ones. As part of the experiments of the next stage, it is proposed to dynamically study the development of the response of clusters of prebiotic microsystems to multi-mode oscillations in parameters in a non-equilibrium environment, including the appearance of induced extremely fast chemical reactions. Through a combination of spontaneous and induced chemical reactions, real proto-biochemical pathways to the emergence of life can be identified. A general approach to experiments of this kind is outlined in [12].
